# A collaborative approach to exercise provision for people with Parkinson’s - a feasibility and acceptability study of the PDConnect programme [version 2; peer review: 2 approved]

**DOI:** 10.12688/amrcopenres.12936.2

**Published:** 2021-04-01

**Authors:** Julie Jones, Lyndsay Alexander, Elizabeth Hancock, Kay Cooper

**Affiliations:** 1 School of Health Sciences, Robert Gordon University, Aberdeen, AB10 7QT, UK; 2 AHP Directorate, NHS Grampian, Aberdeen, UK

**Keywords:** Parkinson’s, exercise, behaviour change, empowerment, partnership

## Abstract

**Background:**

Exercise has been shown to be beneficial for people with Parkinson’s (PwP), slowing the rate of decline of motor and nonmotor symptoms, with emerging evidence associating exercise with a neuroprotective effect. Current exercise provision is time-limited, and delivered in the absence of strategies to support long-term adherence to exercise. With a growing Parkinson’s population, there is a need to develop long-term sustainable approaches to exercise delivery. The primary aim of this study is to assess the feasibility and acceptability of a multicomponent intervention (PDConnect) aimed at promoting physical activity, and self-management for PwP.

**Methods:**

A convergent fixed parallel mixed methods design study will be undertaken. The study aims to recruit 30 PwP, who will be randomly allocated into two groups: (i) the usual care group will receive physiotherapy once a week for six weeks delivered via Microsoft Teams. (ii) The PDConnect group will receive physiotherapy once a week for six weeks which combines exercise, education and behaviour change interventions delivered by NHS Parkinson’s specialist physiotherapists via Microsoft Teams. This will be followed by 12 weekly sessions of group exercise delivered on Microsoft Teams by fitness instructors specially trained in Parkinson’s. Participants will be then contacted by the fitness instructors once per month for three months by video conferencing to support exercise engagement. Primary feasibility data will be collected during the study, with acceptability assessed via semi-structured interviews at the end. Secondary outcomes encompassing motor, non-motor and health and well-being measures will be assessed at baseline, at six, 18, and 30 weeks.

**Discussion:**

This pilot study will establish whether PDConnect is feasible and acceptable to PwP. This will provide a platform for a larger evaluation to assess the effectiveness of PDConnect at increasing exercise participation and self-management within the Parkinson’s Community.

**Trial registration:**

Registered on ISRCTN (ISRCTN11672329, 4^th^ June 2020).

## Introduction

Parkinson’s is the second most common neurodegenerative condition^1^, with global prevalence estimated to exceed 12 million by 2040^2^. Parkinson’s has over 40 recognised motor and non-motor symptoms, which negatively affect function and quality of life (QoL)^3^. Despite major advances in the understanding of Parkinson’s, the cure remains elusive, as do effective long-term interventions. Medical management focusses on limiting the impact of symptoms, but medication has a time-limited effect and is associated with substantial side effects. This has fuelled the need to look at alternative strategies for those living with Parkinson’s. Exercise has been hailed as the new medicine for Parkinson’s and is no longer viewed as a complementary intervention, but is considered of equal importance to medication^3^. Exercise is defined as activities, which are planned, structured, and purposeful, with the intention of improving and/or maintaining one or more components of physical fitness^4^. Exercise is a subcategory of physical activity, the latter being an umbrella term which encompasses bodily movements produced by skeletal muscles. Physical activity includes a wide range of behaviours including gardening, housework and leisure related activities^4^.

**Figure 1:**
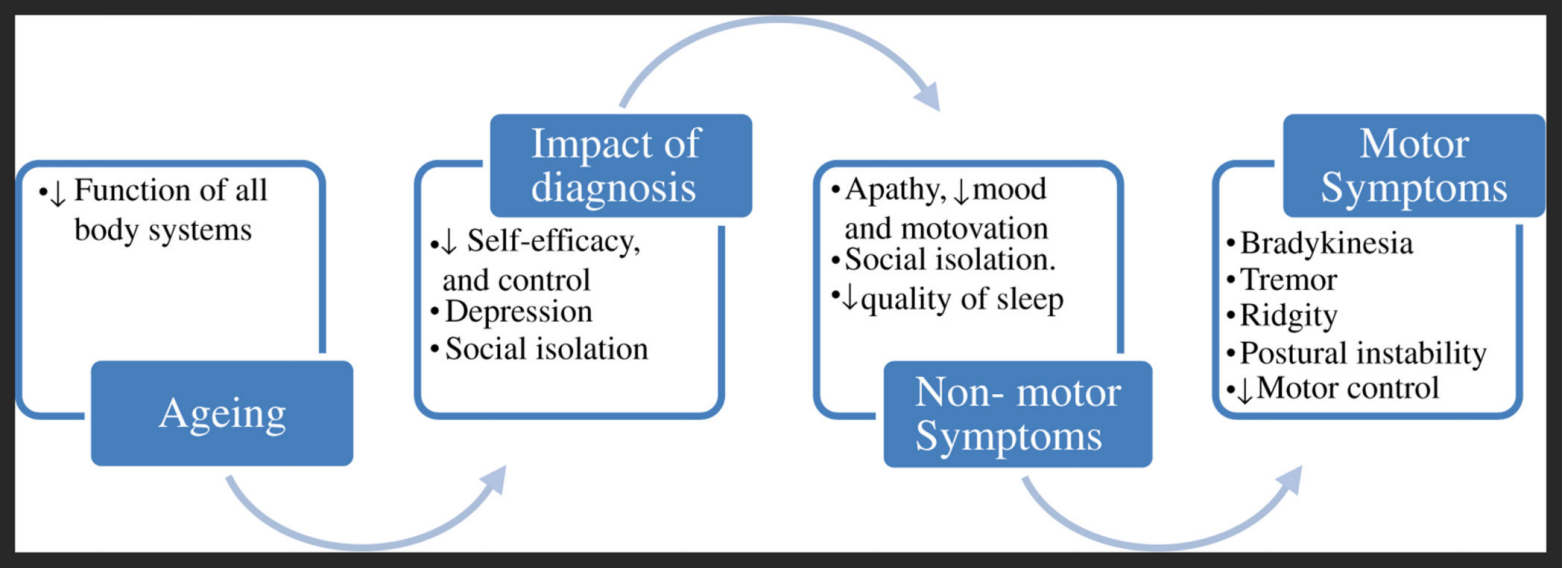
Complex interplay of factors which negatively impact physical activity participation among people with Parkinson’s.

The progressive nature of Parkinson’s^5^ limits participation in exercise and initiates a vicious cycle of inactivity. Inactivity results in deconditioning, leading to reductions in muscle strength, flexibility, balance, aerobic fitness and an increased risk of falls^6^. This is compounded further by non-motor symptoms such as fatigue and depression, which reduce motivation to be active^7^. These factors culminate in reduced socialisation, and subsequently QoL^3,8,9^. The long-term sequela being that the body becomes deconditioned not only as a result of Parkinson’s, but also due to inactivity, as illustrated in Figure 1. Consequently, up to 80% of people with Parkinson’s (PwP) are classed as sedentary^10^, with exercise levels declining from diagnosis^11^. The impact of Covid-19 has further impacted on physical activity levels^12^. Consequently, PWP have reported deterioration in Parkinsonian symptoms, and mental health and well-being due to restrictions put in place due to Covid-19^13^. Regular participation in exercise has been shown to slow down the rate of symptom progression^14–17^ in Parkinson’s; therefore, there is an urgent need for health interventions, which support PwP to adopt long-term exercise habits.

Exercise participation among PwP is associated with improvements in strength, balance, gait, and cognition^6,18,19^ with emerging evidence linking high intensity exercise with a potential disease-modifying effect^17,20–22^. Owing to the diversity of parkinsonian symptoms, sole reliance on any one form of exercise is not advocated; instead a multimodal approach is required^23^. Research suggests that frequency and intensity of exercise are key determinants of successful exercise prescription, with guidelines advocating moderate to high intensity exercise conducted five times a week^24,25^. Moreover, exercise needs to be prescribed for a minimum of ten weeks, to promote the physiological adaptation and skill acquisition required to develop self-confidence with exercise. However, the current healthcare system does not have capacity to support high intensity input^26–28^, nor support exercise long-term, and therefore alternative models to delivery of exercise for PwP are urgently required^29^.

Simply instructing PwP to exercise is not effective^23^. Rather, exercise needs to be delivered as a package of care, which can be delivered long term to support the development of an exercise habit. Healthcare policy proposes that self-management programmes are the mechanism to effectively manage long-term conditions^26^ and support sustained behaviour change.

Self-management evolved from the Chronic Care Model proposed by Wagner *et al*. (1998)^30^, emphasising the importance of organisational change to promote practical, supportive, evidencebased interactions between an informed patient and a proactive practice team. Self-management programmes aim to empower participants through the development of self-efficacy. However, PwP report that access to exercise and ability to self-manage are their greatest unmet needs^31^. Combining exercise with behaviour change and education, as a combined health intervention, has been proposed to be more effective than any one component delivered in isolation^32^. Therefore, delivering exercise as part of a self-management programme embedded with behaviour change strategies could assist the development of exercise self-efficacy, self-actualisation and empowerment of PwP to assume a partnership role within their care.

Telehealth was gaining momentum prior to the Covid-19 pandemic and has seen rapid deployment across many healthcare sectors, including exercise delivery. The role of telehealth, using online means to deliver exercise, may be a practical solution to the evolving challenges of delivering health during the Covid-19 pandemic and beyond, with digital health being central to Scottish government healthcare delivery plans^33^. The potential value of telemedicine as a mechanism to support monitoring of symptoms, and medication management is widely acknowledged^34–36^. PwP have reported high satisfaction with online support^37^, with some preferring online delivery compared with face-to-face^38^. Online delivery offers a flexible means to delivering exercise which can be delivered within the home, removing environmental barriers and mitigating interpersonal barriers, providing exercise in a de-medicalised environment, reducing participant anxiety and costs for providers. As such online delivery has potential to improve access and adherence to exercise, as well as provide a more cost and time efficient mode of delivery. Home-based exercise has been shown to be effective^39^, feasible, and safe even when delivered at moderate to high intensity for PwP^40^. However, use of unsupervised video guided exercise health apps, while feasible among PwP^41^, report poor adherence, high drop-out, with few participants meeting their activity goals. Suggesting that supervision is needed to promote and maintain engagement in exercise when delivered online. However, the feasibility and acceptability of remotely delivered online supervsised exercise for PwP has yet to be fully explored.

Following Medical Research Council (MRC) Guidelines for Designing Complex Interventions^42^, this study aims to explore the feasibility and acceptability of an online collaborative approach to exercise, delivered by specially trained NHS physiotherapists and fitness instructors, which incorporates individualised exercise, education, and behaviour change techniques to support long-term exercise participation for PwP.

## Protocol

Protocol Version: Version 4, 29^th^ January 2021

### Objectives

To determine the feasibility and acceptability of a remotely delivered multi-component intervention (PDConnect) aimed at promoting individualised exercise, physical activity and exercise self-management among community dwelling adults with Parkinson’s.

### Study design

A fixed parallel convergent mixed methods design will be employed.

### Setting

This study will be conducted in North East Scotland delivered exclusively online using Microsoft Teams.

### Participants

**Figure 2:**
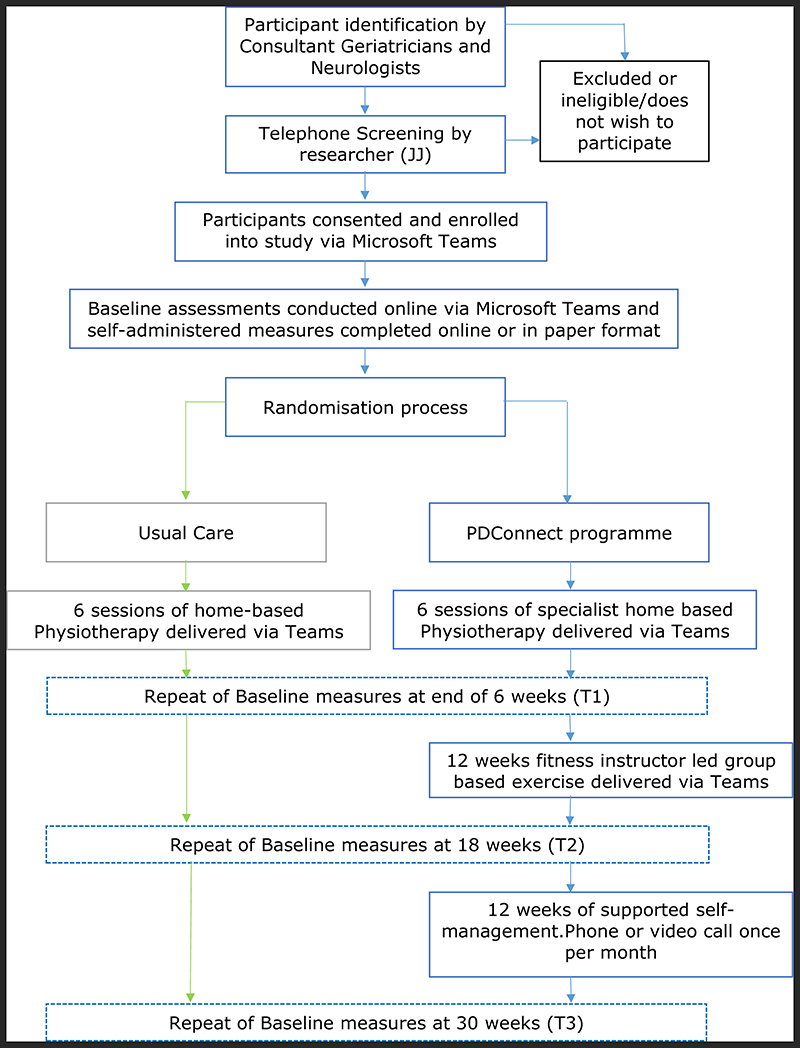
Study flowchart.

This study aims to recruit 30 PwP, which aligns with prior feasibility studies^43,44^ and will allow sufficient numbers for group exercise. Using convenience sampling, recruitment will be carried out in partnership with NHS consultant neurologists and geriatricians. The consultants will identify eligible PwP who meet the study inclusion criteria (Table 1) and will provide them with a participation information sheet *(Extended data* ^45^) and the principal researcher’s contact details. Those contacting the researcher will be given opportunity to ask any further questions and will be screened to ensure safety to exercise. PwP who are considered eligible will be offered an appointment within two weeks to undertake consent and baseline measures within their own home, prior to randomisation to either PDConnect or usual care (Figure 2).

### Randomisation and blinding

Randomisation will be conducted by a Chartered Statistician independent to the study. Stratified random sampling by gender and physical activity will be employed to ensure balance at baseline. Using computer-generated random number sequencing (Excel, Microsoft Corporation) in a ratio of 1:1, a random number will be placed in a sealed, sequentially numbered, opaque envelope. PwP will be allocated randomly by extracting the random number from the envelope and will be notified of group allocation via letter. Group allocation will be concealed from the principal researcher until the end of the study, so that they remain blind to group allocation. Owing to the nature of the intervention being exercise, blinding of PwP is not possible. General Practitioners and Parkinson’s Consultants will be informed of their patients’ involvement within the study.

### Physiotherapist/fitness instructors

Four NHS Physiotherapists will be recruited via purposeful sampling. Eligible Physiotherapists will have a minimum of two-years clinical experience including neurology and geriatrics. The Physiotherapists will be randomly assigned to deliver either PDConnect (n=2) or usual care (n=2), by the use of sealed envelopes provided by the research assistant. Two Fitness Instructors will be recruited from the University Sport facility and will be Register of Exercise Professionals (REPS) accredited (or equivalent). The two Physiotherapists and Fitness Instructors delivering PDConnect will participate in a blended learning training package aimed at developing expertise in exercise prescription for PwP and behaviour change techniques. Those physiotherapists delivering usual care will not receive any training.

### Intervention

#### Usual care

Usual care is defined as physiotherapy delivered by community-based physiotherapists (Band 6 or above) who have not received specialist postgraduate training in treatment and management of Parkinson’s. PwP will receive six one-to-one physiotherapy sessions lasting up to an hour delivered online via Microsoft Teams. Each session will include assessment, treatment, goal setting and intervention delivery, and will be supplemented by a home exercise programme (HEP). Treatment choices will be guided by participant need, as per standard practice.

#### PDConnect

PDConnect is an evidence-informed exercise intervention underpinned by empowerment theory^47^, with the aim of providing PwP with a toolkit of behaviour change techniques (BCTs) to promote participation in exercise and exercise self-management. The PDConnect programme combines specialist physiotherapy, group-based exercise and self-management, with education and behaviour changes strategies threaded throughout. The programme consists of three components: i) six sessions of one-to-one specialist physiotherapy delivered at home; ii) 12 weekly sessions of group-based exercise; iii) 12 weeks of self-management, where participants will be contacted monthly. Each component will be delivered online using Microsoft Teams. To ensure participant safety during PDConnect, all participants will complete a home risk assessment, complete a participation statement, aligning with best practice guidance on online delivery of exercise^48^, and will receive an induction into the use of Microsoft Teams.

##### Specialist physiotherapy

Participants will receive six one-to-one physiotherapy sessions over six weeks, to promote continuity of treatment and develop a foundation to promote behaviour change and self-confidence with exercise engagement. Each one-hour session will encompass exercise, embedded with education and behaviour change interventions, and development of a HEP. BCTs will be will selected from the Behaviour change taxonomy described by Michie *et al*. (2013)^49^. The application of BCTs are mapped to each session in a developmental manner, to support development of exercise self-management. Key BCTs will include: goal planning, shaping knowledge, problem solving, and developing self-awareness, and in the later stages self-belief, reward, and regulation are emphasised. Exercise prescription will encompass strength, aerobic, balance, cognitive, gait, and functional components, with a focus on functional movement patterns, quality and amplitude of movement, in dual and single task activities as per the European physiotherapy guidelines^50^. Participants will be encouraged to work at a moderate to high intensity, defined as “somewhat hard” on the Borg Rating of Perceived Exertion scale^51^. Exercises will be mutually selected and delivered in an educational manner, to promote understanding, motivation and adherence. Throughout the PDConnect intervention exercise will be progressed on an individual basis. Exercises will be prescribed in a progressive manner as advocated by the European Physiotherapy Guideline for Parkinson’s by increasing repetitions, speed, load, or task complexity^50^.

Participants will be supported to develop a weekly exercise activity planner and will record all activity within an activity diary. One-to-one sessions will be supplemented by a HEP aligning with the participant’s treatment goals and exercises will be selected from the REHABGuru (Potters Bar, UK) exercise library. Participants will be encouraged complete their HEP five times a week, for a minimum of 30-minutes, and to record all exercise within their activity diary. A handover sheet will be completed for each participant by the Physiotherapist incorporating summary of physiotherapy, goals, and HEP prior to the participants progressing onto the group based element of PDConnect.

##### 12-week group exercise

Two online exercise groups will be run, with a maximum of eight PwP in a group, aligning with best practice guidelines^48^. To minimise distractions, and reduce safety risk while exercising, participants will also be advised to have their Teams screen set at speaker view allowing them to see only themselves and the fitness instructor. When providing individual feedback during the class the “spotlight” feature within Microsoft Teams will be utilised so only the participant and the fitness instructor are visible on screen.

Family and carers will also be invited to attend, following basic health screening acknowledging the value of social support reported by PwP. Based on current research, the class will run for a minimum of 60-minutes. This will encompass, 10 minute warm up and cool down and ten exercise stations, spending four minutes at each station^4^. Participants will undertake the same exercises at the same time. Each station will have four levels of difficulty, tailored to individual ability. Informed by Parkinson’s guidelines, exercises will include; mobility, strengthening, aerobic, balance, cognitive, goal-oriented components, with an emphasis on large amplitude movements and intensity of effort. All participants will receive an introduction to each exercise, encompassing key teaching points, practical demonstration, proposed benefits and purpose of the exercise. Videos of each exercise and individual levels will be available on Microsoft Teams to prior to participating. Throughout the class, the fitness instructor will be present to provide feedback, refine technique and progress participants as appropriate. The remaining 30-minutes of the class is allocated to group-based discussion facilitated by the fitness instructor. During this time participants will be able to see and interact with each other and the fitness instructor on screen. The purpose of these discussions is to promote shared experience and learning. Participants will be encouraged to continue with their HEP, and these will be reviewed every three weeks and progressed as required by the fitness instructor.

##### 12 weeks self-management

During this phase participants will exercise independently, following the HEP which has been developed during the programme. Participants will receive a 20-minute phone or video call every month from the fitness instructor, to review and adapt the HEP as required and to support problem solving to overcome any barriers that may have arisen.

### Outcome measurement

#### Primary outcome measurement

A mixed methods approach will be adopted to establish feasibility, acceptability and fidelity. As such, data will be collected on feasibility and acceptability as outlined below:

##### Feasibility

Feasibility will be assessed by collecting data on the following:

- Recruitment and retention rates
- Adherence rates
- Time required to recruit to target
- Number of eligible participants required to recruit target sample size
- Number of PwP who complete each aspect of PDConnect
- Feasibility of testing procedures and data collection methods, including completion rates of outcome measures

##### Acceptability

Prospective acceptability (reasons for not taking part/discontinuation or dropping out) will be assessed via a survey. Semi-structured interviews will be conducted on completion of the study to explore the potential motivators and barriers associated with the PDConnect programme and experiences of those participating within the study (i.e. physiotherapists, fitness instructors and PwP). Interviews will be conducted by the principal researcher, using Microsoft Teams video conferencing. Interviews will be conducted following the approached outlined by Creswell and Plano-Clark (2011)^52^, adopting a topic guide *(Extended data* ^45^) to inform conduct of the interviews. Interviews will be recorded via digital voice recorder and fully transcribed after the event. A fieldwork journal will also be kept by the principal researcher when undertaking the interviews to note additional relevant details which arise. All transcripts will be sent to interviewees following the interview, to confirm accuracy.

##### Fidelity assessment

A random sample of six physiotherapy interventions and three group exercise sessions will be videoed to facilitate fidelity assessment. The focus of this assessment will to establish whether the intervention is delivered as planned, in particular content, delivery and duration. Fidelity assessment will be conducted by the researcher following completion of the study after unblinding.

#### Secondary outcome measurement

Although this study is not powered to detect statistically significant changes in clinical outcome measures, secondary outcomes (measured at baseline, at six, eighteen and thirty weeks) will be collected in order to provide some preliminary data on outcomes such as physical activity, motor and non-motor symptoms, depression and anxiety, fatigue, function, self-efficacy and quality of life. This will generate data on effect sizes to inform sample size calculation for a future effectiveness study. A suite of outcome measures will be employed in order to inform selection of the most appropriate measures for use in a future effectiveness study. All physical measures will be conducted by the principal researcher who is blind to the intervention allocation. Physical measures will be conducted using Microsoft Teams, with participants sent self-administered questionnaires in the post or online depending upon preference. Due to ongoing Covid restrictions, undertaking of physical measures face to face physical measures are not possible. Therefore, to maintain participant safety some physical measures will be omitted due to the online mode of delivery of this study imposed by Covid. This includes the Functional Gait Assessment, 6 minute walk test and the 10 meter walk test.

##### Motor function will be measured by

Unified Parkinson’s Disease Rating Scale (UPDRS), Schwab and England, and Activities specific Balance Confidence Scale. All measures are recommended by the Movement Disorders Task Force^46,53,54^ and have recognised psychometric properties for PwP. In addition, self-physical activity in particular step count, will be measured by the use of wrist-worn activity tracker (Mi band version 5.0). The Mi band has good internal consistency during the six-minute walk test and stairs climb [ICC: 0.83]^55^. Daily step counts will be recorded within an activity diary.

##### Non-motor function will be measured by

UPDRS, Lille Apathy scale, Parkinson’s Anxiety scale, Parkinson’s Fatigue Scale, Geriatric depression scale, Parkinson’s disease questionnaire 39, Nottingham Health Profile. All these measures are recommended by the Movement Disorders Task Force^56–60^ and have recognised psychometric properties for People with Parkinson’s. In addition, the self-efficacy scale for exercise, which has been used widely in long-term conditions, and the Warwick Edinburgh Mental Health and Wellbeing Scale, which has established reliability for use in the general population^61^, will be used. The total estimated to complete all measures is 90 minutes.

### Planned data analysis

As this is an acceptability and feasibility study, the analysis will focus on the key parameters necessary for conducting a future trial. Most of the analysis will be descriptive in nature. The principal researcher will intelligently transcribe qualitative data from the semi-structured interviews. Participants will be sent completed transcribed documents to check for accuracy and make amendments or additions as required. Data will be analysed using NVivo version 12; following a framework analysis approach^62^ Coding and categorisation of data will be reviewed by a second researcher. Quantitative data including feasibility measures, demographic information, and baseline measures will be analysed using simple descriptive statistics using SPSS. Preliminary data will also be analysed to estimate the size of effect, by analysing the difference of mean change between PD Connect and Standard care at set points of six, 18 and 30 weeks. This will be calculated for each quantitative outcome measure (e.g. step count as measured by the fitness tracker, strength, balance, UPDRS, QoL) and will be established at each time point in order to calculate effect sizes and inform future sample size calculation. Further, this will inform the primary outcome for use in the future effectiveness study.

### Monitoring

Analysis will be undertaken at set points during the study, following baseline measurements, and after the six, 18 and 30 weeks measurements are conducted. In a review of Parkinson’s clinical trials, Allen *et al*. (2012) reported that 69% of interventions retained 85% or more of their participants. Owing to the small sample size in the current feasibility study (n=30), an 85% retention rate has been set within the PDConnect study, with retention rate below this figure deemed as criterion for not progressing to a full trial. The target adherence for this study is set at 77% allowing participants to miss one physiotherapy session, and a maximum of three out of 12 weeks of group-based exercise. With exercise participation there is a potential falls risk. Within the activity dairy participants randomised to either arm of the study are asked to note all falls within their activity diary and contact the researcher directly to report any falls. A priori, more than five people reporting a falls during the intervention would trigger referral to the Data Management Committee (DMC) to discussion potential for study cessation. The DMC for this study aligns with the National Institute for Health Research Guidelines. The DMC will consist of four people who are independent to the study and institution where this study is being hosted. This will include a statistician, a Parkinson’s specialist clinician, a senior researcher, and a person with Parkinson’s. Meetings will be held annually, or more frequently if required, with a minimum quoracy for all meetings to be 67% (two thirds) of appointed members. The study will be monitored by all members of the project management group. They will oversee the conduct of the trial. As the sponsor, Robert Gordon University may request to audit processes and procedure, as will NHS Grampian who provide Research and Development (R&D) site approval for this study.

### Ethics and dissemination

The study was approved by the Liverpool Central REC Centre (IRAS Number 280159) and has local NHS Grampian Research and Development approval (2020RG001E). Any protocol modifications required will be communicated by the principal researcher to the ethics and R&D departments, and updated on ISRCTN and reported in publications arising from the study. All participants will be asked to provide signed informed consent *(Extended data* *^45^)* to participate in this study. Consent will be taken by the principal researcher in the participant’s home. Participation in this study will not negate referral to ancillary services such as occupational therapy, should an identified need arise. Any need for compensation will be addressed through the sponsors insurance. All data will be stored in line with GDPR guidelines. Project data, and materials sent for publication, will be anonymised by removing statements identifying participants. The data will be stored securely in a password-protected research drive within the research teams’ institution accessible only to the research team. Only the research team will have access to the final data set, and acknowledging this is a feasibility study, data will not be publicly available after completion of the study. The anonymised findings will be included in the first author’s doctoral thesis as well as being disseminated through peer-reviewed academic journals, national and international conferences and public events.

## Discussion

Exercise has been shown to slow down the rate of progression of Parkinson’s. However, a significant proportion of PwP remain inactive. Current exercise programmes are delivered short term, in isolation and fail to provide PwP the strategies and skills to support long term sustained behaviour change. PDConnect is an innovative intervention combining self-management, and exercise, fostering a collaborative approach between physiotherapy, fitness instructors and PwP. This combination may promote long-term exercise adherence amongst PwP by providing them with the required skills, knowledge and abilities to participate in exercise. This feasibility and acceptability study will determine recruitment and retention rates, testing the procedures and exploring the acceptability of the programme as well as investigating potential changes in motor, non-motor and health and wellbeing outcomes. This study is in line with the MRC guidance for developing complex interventions (testing procedures, estimating recruitment and retention, determining sample size).

The disparity in intervention volume and provision of training to staff delivering the intervention arm is a recognised confounder. However, the study is comparing PDConnect (a novel combination of specialist physio, exercise & self-management) with usual care, therefore the need for specialist training is required. As this study is a feasibility and acceptability study, findings will be used to refine the proposed intervention within the future RCT, which will include a process evaluation in the RCT to explore assumptions associated with this complex intervention. This study will not be able to determine effectiveness, but will inform whether PDConnect is feasible to deliver and acceptable to PwP.

The quantitative data will provide an initial understanding of the potential benefits of PDConnect, with qualitative data serving to increase understanding of the perceptions of those participating within PDConnect. The results will indicate the feasibility and acceptability of the intervention, which will inform modifications to the intervention, processes and procedures prior to embarking on a larger scale study, in order to investigate the full effectiveness and cost-effectiveness of the programme.

### Trial information

This study was registered on ISRCTN (ISRCTN11672329, 4^th^ June 2020).

Trial sponsor is Vice-Principal for Research and Commercialisation, c/o Jill Johnston, Robert Gordon University, Aberdeen (j.johnston4@rgu.ac.uk).

### Study status

This study has been awarded full Ethical and Research and Development Approval, and has commenced recruitment.

## Supplementary Material

Full Text XML

manifest

meta

Supplement

## Figures and Tables

**Table 1:** Study inclusion criteria.

Inclusion criteria	Exclusion criteria
Confirmed diagnosis of Parkinson’s by Parkinson’s specialist Consultant Neurologists and Geriatricians Stage I-III Hoehn and Yahr Scale fluctuation Mild to severe gait disturbance with a score of <2 on the Unified Parkinson’s disease Rating Scale (UPDRS) item 29^46^ Able to walk independently with or without a walking aid further than 100m Stable medication for more than three weeks Able to speak and understand English without assistance Own or have access to a laptop or tablet with a webcam, which is compatible with Microsoft Teams.	Secondary or atypical Parkinsonism Severe, unpredictable episodes of motor Use of medications known to interfere with cognitive function History of neurological diseases other than Parkinson’s Any unstable mental or physical condition that prevent consenting and participating in exercise. Unstable or uncontrolled medical conditions

## Data Availability

Underlying data No underlying data are associated with this article.

## revised : Amendments from Version 1

Clarification and additional detail in relation to the intervention has been added. In particular, the behavioural change approach adopted within the study, and how exercise will be progressed during the course of the intervention. Further clarity is also provided on how the group exercise element will address individual participant needs, and ensure participant safety. Owing to ongoing Covid restrictions, this new version also reflects updated and ethically approved changes to the outcome measurement section within the protocol.

**Any further responses from the reviewers can be found at the end of the article**
