# Supplementary material for: A collaborative approach to exercise provision for people with Parkinson’s - a feasibility and acceptability study of the PDConnect programme [version 2; peer review: 2 approved]
Source: AMRC Open Res. Author manuscript; Available in PMC 2021 Jun 15. (PMC7610987; doi:10.12688/amrcopenres.12936.2)
Supplement: Supplement [file EMS126665-supplement-Supplement.pdf]

Open Peer Review

Current Peer Review Status: ✔ ✔

Version 2

Reviewer Report 21 April 2021

https://doi.org/10.21956/amrcopenres.14059.r26662

© 2021 Morris R. This is an open access peer review report distributed under the terms of the Creative Commons Attribution License, which permits unrestricted use, distribution, and reproduction in any medium, provided the original work is properly cited.

✔

Rosie Morris
Department of Sport, Exercise, and Rehabilitation, Northumbria University, Newcastle upon Tyne, UK

The revisions have improved the article and the reviewer has no further comments.

Competing Interests: No competing interests were disclosed.

Reviewer Expertise: Ageing, Neurodegenerative disease, Physiotherapy, mobility (gait, balance, falls), cognition

I confirm that I have read this submission and believe that I have an appropriate level of expertise to confirm that it is of an acceptable scientific standard.

Reviewer Report 08 April 2021

https://doi.org/10.21956/amrcopenres.14059.r26663

© 2021 Allen N. This is an open access peer review report distributed under the terms of the Creative Commons Attribution License, which permits unrestricted use, distribution, and reproduction in any medium, provided the original work is properly cited.

✔

Natalie Allen
Discipline of Physiotherapy, Faculty of Medicine and Health, University of Sydney, Sydney, Australia

The authors have addressed all my comments. The protocol is clearly written and detailed.

Competing Interests: No competing interests were disclosed.

Reviewer Expertise: My research focuses on exercise and physiotherapy-based interventions for people with Parkinson's disease.

I confirm that I have read this submission and believe that I have an appropriate level of expertise to confirm that it is of an acceptable scientific standard.

Version 1

Reviewer Report 29 January 2021

https://doi.org/10.21956/amrcopenres.14007.r26601

© 2021 Morris R. This is an open access peer review report distributed under the terms of the Creative Commons Attribution License, which permits unrestricted use, distribution, and reproduction in any medium, provided the original work is properly cited.

?

Rosie Morris
Department of Sport, Exercise, and Rehabilitation, Northumbria University, Newcastle upon Tyne, UK

The study by Jones *et al* aims to establish whether their intervention study, PDConnect, is feasible and acceptable in a cohort of patients with Parkinson's disease. The aim of this intervention is to promote both physical activity and self-management for exercise for these patients. This is an interesting study and timely given the current pandemic. The protocol is clear and well written, there are a number of minor points that could be addressed before indexing.

Abstract
The abstract is clear and concise. The word 'limiting' in 'limiting the rate of decline...' may want to be changed to 'slowing', but this is a minor point.

Introduction
The introduction of the manuscript is comprehensive and provides a good background to the study. There are a few minor points the authors could improve on:

- Throughout the introduction there is discussion around Covid-19 and the influence this has had on face-to-face interventions. Covid-19 has provided a catalyst for seeking virtual interventions; however, the importance of virtual interventions should be recognised further than Covid-19. For example, the need for this treatment for those who live remotely, reduced costs, reduced time required, increased retention rates etc.

- Figure 1 is a little confusing. At present it suggests that impact of diagnosis influences NMS and NMS influence motor symptoms. Perhaps this figure could be altered to represent that the four factors separately impact negatively on physical activity participation.

Protocol

- In the inclusion criteria, does confirmed diagnosis of PD include meeting UK Brain Bank Criteria?

- Within the criteria, could the authors also include H&Y IV, is there a rationale for removing these participants?
- Spelling error within Hoehn and Yahr under 'Randomisation and Binding'
- Within the project protocol it is highlighted that specialist Physiotherapists with training will be involved in the PDConnect group but not the usual care group. As the main outcome of the study is addressing self-management, does this require those specialist skills? Perhaps the difference in training may influence the outcomes of the study
- The protocol states that exercise needs to be completed five times per week. Within the introduction there is a suggestion that daily exercise is required. It is appreciated that it would be difficult to retain participants if daily exercise was expected. But does this need to be addressed within the self-management programme?
- Within the outcome measures, please state which sections of the UPDRS will be utilised for both motor and non-motor symptoms and whether you are using the MDS-UPDRS or the UPDRS
- It may be worth including cognitive assessments within the non-motor symptoms outcome measures due to the benefit of exercise on cognition
- An activity tracker is going to be used to monitor physical activity. Which monitor is going to be used and is there a reference for validity and reliability?

Is the rationale for, and objectives of, the study clearly described?
Yes

Is the study design appropriate for the research question?
Yes

Are sufficient details of the methods provided to allow replication by others?
Partly

Are the datasets clearly presented in a useable and accessible format?
Yes

Competing Interests: No competing interests were disclosed.

Reviewer Expertise: Ageing, Neurodegenerative disease, Physiotherapy, mobility (gait, balance, falls), cognition

I confirm that I have read this submission and believe that I have an appropriate level of expertise to confirm that it is of an acceptable scientific standard, however I have significant reservations, as outlined above.

Reviewer Report 13 January 2021

https://doi.org/10.21956/amrcopenres.14007.r26584

© **2021 Allen N.** This is an open access peer review report distributed under the terms of the Creative Commons Attribution License, which permits unrestricted use, distribution, and reproduction in any medium, provided the original work is properly cited.

?

Natalie Allen

Discipline of Physiotherapy, Faculty of Medicine and Health, University of Sydney, Sydney, Australia

This protocol is for a randomised, mixed methods study exploring the acceptability and feasibility of a multicomponent intervention aimed at increasing physical activity and improving exercise self-management in people with mild to moderate Parkinson's disease (PD). The benefits of exercise for people with PD are well established, yet most people with PD do not perform the recommended amount. Therefore, this project addresses the important issue of how to establish and support long term exercise participation amongst people with PD. The project intervention is delivered via telehealth, and therefore complies with limitations due to the Covid-19 pandemic, as well as providing important information about the acceptability and feasibility of telehealth that can be used in the future (e.g., for people living in rural and remote areas, or who are unable to travel). The project is designed to provide the information required to design an appropriately powered randomised controlled trial exploring the effectiveness of the intervention.

General comments
The concept of self-management is clearly explained and justified; however, the self-management component of the program appears to be about exercise self-management (rather than self-management more broadly). I suggest this is directly clarified in the text (e.g., by using the term 'exercise self-management' where applicable.
Some abbreviations did not appear to be written in full on the first use – eg PWP, MRC

Abstract
The abstract is written clearly and contains the key information.

Introduction
The introduction sets up the rationale for the project and is written in a clear and logical sequence. However, there are two sentences near the end of the second last paragraph that are written poorly and should be revised for clarity and English expression (starting from "The use of a video guided exercise health app…")
Please consider revising Figure 1. What do the arrows represent? To me, the arrows suggest that one thing is leading to or causing the other, but in this figure this is not necessarily the case. For example, non-motor symptoms do not lead to or cause motor symptoms. The figure legend and associated information in the text suggest the figure shows how these factors influence physical activity, however this is not clear in the figure itself.

Protocol
This is a complex intervention, and therefore I expect it is difficult to clearly describe. The research

protocol in the extended data is very helpful – I suggest referring to this document in the main text, as has been done for the topic guide, consent form etc. While the overall design is clear, and a research protocol is provided in the extended data, there are some details that I think should be clarified in the main text.

- Please describe how the BCTs will be embedded into the specialist physiotherapy intervention. The BCTs are not referenced and it appears that some are from the Behaviour Change Taxonomy while others do not follow taxonomy wording (e.g., developing self-awareness). I suggest using wording from the Behaviour Change Taxonomy and referring to the taxonomy.

- Will exercise be progressed during the program? If so, how?

- How will the group exercise sessions work? How many people will be in any one group? Will participants be able to see each other and/or the instructor? If so, this could be distracting and raise safety concerns – how will this be managed? How will the exercise level be determined? Will participants be told their level and exercises so they can review the resources on teams prior to the class? Will participants all be doing the same exercise at the same time?

- Will there be communication regarding specific participants between the physiotherapist and fitness instructor? How will this be organised? Will the home exercise program used in the 12 weeks of exercise self-management be the same as that developed during the specialised physiotherapy?

Figure 2 – consider adding further information to clarify the location/method of delivery. For example, indicate that consent and assessments will occur in participant's homes via an in-person visit, and that the physiotherapy, fitness instructor exercise group and supported exercise self-management will be using videoconferencing.

Randomisation and blinding: the word 'Yahr' has a typo
The level of expertise of the physiotherapists is described in a way that is specific to the UK – I suggest revising this to describe it in a way that an international audience will understand.
The volume of intervention in the usual care group is less than the volume of intervention in the PDConnect group. This is likely to influence the secondary outcome measures and should be taken into account when using the secondary outcome results to design and power a large-scale RCT.

The PDConnect program is underpinned by empowerment theory – please provide a reference for empowerment theory.
The information about the time required for the 12-week group exercise class is inconsistent. The class is to be a minimum of 60 minutes; however, it includes 40 minutes of exercise and 30 minutes of discussion.

Outcome measurement
Acceptability - The sentence stating that "Interviews involving those delivering the intervention will be conducted using Microsoft Teams" appears to be superfluous.
Will field notes or journaling be used alongside the semi-structured interviews?
What will the fidelity assessment be looking for specifically?

Motor function and non-motor function are to be measured using the UPDRS. Are you using the

old UPDRS, or the newer, MDS-UPDRS version? A reference would also help to clarify this.
In the motor function measurement section Please specify which activity tracker you will be using, and what the tracker will be measuring (i.e., steps). Is there a justification for wearing the tracker on the wrist when this is likely to overestimate physical activity?

Monitoring: the authors note that if more than 5 people report falls, then the data management committee will discuss possible study cessation. Is this any fall, or a fall while undertaking the prescribed exercises? Please clarify. People with PD are known to be at a high risk of falls; therefore, I consider it likely that more than 5 out of 30 people will fall during the course of the study – though hopefully not while undertaking the intervention.
Who are the project management group?

Is the rationale for, and objectives of, the study clearly described?
Yes

Is the study design appropriate for the research question?
Yes

Are sufficient details of the methods provided to allow replication by others?
No

Are the datasets clearly presented in a useable and accessible format?
Yes

Competing Interests: No competing interests were disclosed.

Reviewer Expertise: My research focuses on exercise and physiotherapy-based interventions for people with Parkinson's disease.

I confirm that I have read this submission and believe that I have an appropriate level of expertise to confirm that it is of an acceptable scientific standard, however I have significant reservations, as outlined above.

Comments on this article

Version 1

Author Response 19 Mar 2021
Julie Jones, Robert Gordon University, Aberdeen, UK

“Thank you for your comments and feedback in relation to this study protocol.   The feedback to each comment is written in italics below to that the responses can be easily distinguished from the reviewer’s comments”.

Rosie Mearns comments
Abstract
The abstract is clear and concise. The word 'limiting' in 'limiting the rate of decline…' may want to be changed to 'slowing', but this is a minor point.
"Thank you for this suggestion, we have changed the wording, encompassing slowing"

Introduction
The introduction of the manuscript is comprehensive and provides a good background to the study. There are a few minor points the authors could improve on:
Throughout the introduction there is discussion around Covid-19 and the influence this has had on face-to-face interventions. Covid-19 has provided a catalyst for seeking virtual interventions; however, the importance of virtual interventions should be recognised further than Covid-19. For example, the need for this treatment for those who live remotely, reduced costs, reduced time required, increased retention rates etc.
"The introduction text has been amended to reflect that Telehealth was gaining momentum prior to the Covid-19 pandemic and has seen rapid deployment across many healthcare sectors, including exercise delivery".

Figure 1 is a little confusing. At present it suggests that impact of diagnosis influences NMS and NMS influence motor symptoms. Perhaps this figure could be altered to represent that the four factors separately impact negatively on physical activity participation
"*A new figure 1 has been submitted in version 2 of the article*"

Protocol
In the inclusion criteria, does confirmed diagnosis of PD include meeting UK Brain Bank Criteria?
"*We have not used the UK Brain Bank Criteria – instead, diagnosis is made by Parkinson's specialist reflecting local clinical practice. This has been clarified within the inclusion criteria*"

Within the criteria, could the authors also include H&Y IV, is there a rationale for removing these participants?
"Rationale for removing stage IV, was to ensure safety with exercising remotely at home. At level four they may or may not be able to stand unassisted, and for safety reasons they were excluded."

Spelling error within Hoehn and Yahr under 'Randomisation and Binding'
"Thank you for highlighting this is now corrected"

Within the project protocol it is highlighted that specialist Physiotherapists with training will be involved in the PDConnect group but not the usual care group. As the main outcome of the study is addressing self-management, does this require those specialist skills? Perhaps the difference in training may influence the outcomes of the study
"*The provision of training to only those delivering PDConnect is a recognised potential confounder but that the study is comparing PDConnect (a novel combination of specialist physio, exercise & self-management) with usual care. Therefore, training is required for the specialist PT element. AS this is a feasibility and acceptability study, the finding will be used to refine the proposed intervention theory following this study and include a process evaluation in the RCT to explore assumptions associated with this complex intervention".*

The protocol states that exercise needs to be completed five times per week. Within the introduction there is a suggestion that daily exercise is required. It is appreciated that it would be difficult to retain participants if daily exercise was expected. But does this need to be addressed within the self-management programme?
"Thank you for highlighting this. Participants will be encouraged to develop exercise habit will be addressed as part of the self-management, and use of BCTs. This will include structured exercise five times week, with promotion of a physically active lifestyle in addition to this. A minor amendment to the introduction have been made to enhance clarity of this".

Within the outcome measures, please state which sections of the UPDRS will be utilised for both motor and non-motor symptoms and whether you are using the MDS-UPDRS or the UPDRS
"Guidance was taken from local consultants, with the UPDRS being used owing to the costs and licencing with MDS UPDRS. This has been clarified within the referencing with the version by Fahn et al."

It may be worth including cognitive assessments within the non-motor symptoms outcome measures due to the benefit of exercise on cognition
"Thank you for the suggestion, we agree that the benefit of exercise on cognition is important and did consider this when selecting outcomes. However, following discussion with the study team and advisors, due to the battery of outcomes included and the inclusion of cognition within the UPDRS, we decided not to include a specific cognitive measure"

An activity tracker is going to be used to monitor physical activity. Which monitor is going to be used and is there a reference for validity and reliability?
"*Type of tracker, reliability and validity have been added to the manuscript within the section on motor function measures*".

"Thank you for your time taken to review this article and we appreciate your feedback".

NATALIE ALLEN COMMENTS
"Thank you for your comments and feedback, very much appreciated."

General comments
The concept of self-management is clearly explained and justified; however, the self-management component of the program appears to be about <u>exercise</u> self-management (rather than self-management more broadly). I suggest this is directly clarified in the text (e.g., by using the term 'exercise self-management' where applicable.
" *The term exercise self-management has been adopted within the manuscript*"

Some abbreviations did not appear to be written in full on the first use – eg PWP, MRC
"*This has been rectified, thank you".*

Abstract
The abstract is written clearly and contains the key information.

Introduction
The introduction sets up the rationale for the project and is written in a clear and logical sequence. However, there are two sentences near the end of the second last paragraph that are written poorly and should be revised for clarity and English expression (starting from "The use of a video guided exercise health app…")
"This has been amended, thank you".

Please consider revising Figure 1. What do the arrows represent? To me, the arrows suggest that one thing is leading to or causing the other, but in this figure this is not necessarily the case. For example, non-motor symptoms do not lead to or cause motor symptoms. The figure legend and associated information in the text suggest the figure shows how these factors influence physical activity, however this is not clear in the figure itself.
A new figure 1 has been submitted in version 2 of the article"

Protocol
This is a complex intervention, and therefore I expect it is difficult to clearly describe. The research protocol in the extended data is very helpful – I suggest referring to this document in the main text, as has been done for the topic guide, consent form etc. While the overall design is clear, and a research protocol is provided in the extended data, there are some details that I think should be clarified in the main text.
"The research protocol is now referred to in the main text within the amended submission".

Please describe how the BCTs will be embedded into the specialist physiotherapy intervention. The BCTs are not referenced and it appears that some are from the Behaviour Change Taxonomy while others do not follow taxonomy wording (e.g., developing self-awareness). I suggest using wording from the Behaviour Change Taxonomy and referring to the taxonomy.
"Specific Taxonomy has now been referenced within the manuscript, thank you for highlighting this omission. The wording has been adjusted so it aligns with the terminology used within the behaviour change taxonomy within the manuscript, so it is clearer for the reader"

Will exercise be progressed during the program? If so, how?
"Yes, exercise will be progressed within the programme. This will be done on an individual basis, to ensure that all exercise is progressive in nature following the guidelines outlined in the European Physiotherapy Guidelines for Parkinson's".

Text has been added within the manuscript to clarify this point you have raised.
How will the group exercise sessions work? How many people will be in any one group? Will participants be able to see each other and/or the instructor? If so, this could be distracting and raise safety concerns – how will this be managed? How will the exercise level be determined?
"There will be a maximum of 8 PwP in a group, aligning with best practice guidelines for safe delivery of online exercise. The manuscript has been edited to reflect this. Participants will be able to see each other on screen using Microsoft teams. However during classes, participants would be advised to have their screen on speaker view, so they can focus on the instructor and not be distracted by other participants. The PI will have conducted an online home risk assessment and completed a participant information statement prior to commencing the study. This has been added to the manuscript."

Will participants be told their level and exercises so they can review the resources on teams prior to the class? Will participants all be doing the same exercise at the same time?
"Yes, participants will be told their levels and videos of the exercises will be available on the Microsoft teams site prior to the class. All participants will all be doing the same exercise as the same time, however the level of that exercise may differ depending on individual ability. These points have been clarified with the amended version of the manuscript."
Will there be communication regarding specific participants between the physiotherapist and fitness instructor? How will this be organised? Will the home exercise program used in the 12 weeks of exercise self-management be the same as that developed during the specialised physiotherapy?
"Thank you for highlighting this. A summary document will be used including a summary of PT, HEP, participants current goals. This will be saved on a closed channel (ie so participants cannot access this) within the PDConnect Microsoft Teams area. The manuscript has been edited to reflect this. The HEP developed in the 6 weeks of PT will be continued and developed during the 12 weeks group based element of the programme."

Figure 2 – consider adding further information to clarify the location/method of delivery. For example, indicate that consent and assessments will occur in participant's homes via an in-person visit, and that the physiotherapy, fitness instructor exercise group and supported exercise self-management will be using videoconferencing.
"Figure 2 has been updates to reflect your comments"

Randomisation and blinding: the word 'Yahr' has a typo
"This has been corrected, thank you."

The level of expertise of the physiotherapists is described in a way that is specific to the UK – I suggest revising this to describe it in a way that an international audience will understand.
"Thank you, this has been amended, to two years post qualify experience."

The volume of intervention in the usual care group is less than the volume of intervention in the PDConnect group. This is likely to influence the secondary outcome measures and should be taken into account when using the secondary outcome results to design and power a large-scale RCT.
"This factor has been mentioned within the discussion section of the discussion section. Similarly with the above point made in relation to specialist training of the physiotherapist, this will be further considered when designing the future RCT."

The PDConnect program is underpinned by empowerment theory – please provide a reference for empowerment theory.
"Added within manuscript"

The information about the time required for the 12-week group exercise class is inconsistent. The class is to be a minimum of 60 minutes; however, it includes 40 minutes of exercise and 30 minutes of discussion.
"Thank you for highlighting this inconsistency. Participants will engage in 10 stations of exercise each lasting 4 mins, and there will be an additional 10 minutes for warm and cool down, prior to the 30 minute discussion. This will make up the 90 Minute session. This has been clarified within the

manuscript."

Outcome measurement
Acceptability - The sentence stating that "Interviews involving those delivering the intervention will be conducted using Microsoft Teams" appears to be superfluous.
"The word has been removed, thank you."

Will field notes or journaling be used alongside the semi-structured interviews?
"Yes, journal notes will also be used alongside the semi structured interviews, this has been added to the manuscript."

What will the fidelity assessment be looking for specifically?
"Fidelity assessment is to ascertain that the intervention is being delivered as planned, in particular the duration of time spent exercising, content and the style of delivery. Further clarification on this has been added to the manuscript"

Motor function and non-motor function are to be measured using the UPDRS. Are you using the old UPDRS, or the newer, MDS-UPDRS version? A reference would also help to clarify this.
"Reference has been added to the manuscript to clarify this point, thank you."

In the motor function measurement section Please specify which activity tracker you will be using, and what the tracker will be measuring (i.e., steps). Is there a justification for wearing the tracker on the wrist when this is likely to overestimate physical activity?
"The Mi band tracker will be used. The name of this tracker and it reliability and validity have been added to the transcript. Although wrist worn tracker are known to be less reliable. They were selected over LL or body worn devices due to participant convenience"

Monitoring: the authors note that if more than 5 people report falls, then the data management committee will discuss possible study cessation. Is this any fall, or a fall while undertaking the prescribed exercises? Please clarify. People with PD are known to be at a high risk of falls; therefore, I consider it likely that more than 5 out of 30 people will fall during the course of the study – though hopefully not while undertaking the intervention.

"Only falls during the intervention will be considered, the manuscript has been amended to reflect this".

Many thanks indeed for the time taken to review this article and for all the constructive comments, this has been much appreciated."

Competing Interests: Nil
